# Anti‐Inflammatory Compounds From Roots of *Heracleum sphondylium* subsp. *cyclocarpum*

**DOI:** 10.1111/cbdd.70044

**Published:** 2025-02-13

**Authors:** Ekin Kurtul, Özlem Bahadır Acıkara, Büşra Karpuz Ağören, Esra Küpeli Akkol

**Affiliations:** ^1^ Department of Pharmacognosy, Faculty of Pharmacy Zonguldak Bülent Ecevit University Zonguldak Turkey; ^2^ Department of Pharmacognosy, Faculty of Pharmacy Ankara University Ankara Turkey; ^3^ Department of Pharmacognosy, Faculty of Pharmacy Başkent University Ankara Turkey; ^4^ Department of Pharmacognosy, Faculty of Pharmacy Gazi University Ankara Turkey

**Keywords:** anti‐inflammatory activity, Apiaceae, bioactivity‐guided fractionation, byakangelicin, coumarin, heraclenol, *Heracleum sphondylium*, meranzin hydrate

## Abstract

*Heracleum sphondylium* subsp. *cyclocarpum* (K. Koch) P.H. Davis from *Heracleum* L. genus, which is one of the widest genera of the Apiaceae family and known as “Hogweed or Tavşancılotu”. This genus has a variety of traditional uses, including treating gastrointestinal, cardiovascular, gynecological, and cognitive disorders, skin problems, rheumatism, and inflammation. In particular, these plants are commonly used for inflammatory diseases. This research aimed to examine in detail the anti‐inflammatory properties of *Heracleum sphondylium* subsp. *cyclocarpum* roots. Bioactivity‐guided fractionation was used to isolate the active compounds. Carrageenan‐ and prostaglandin E2‐induced inflammation models were employed to test the activity. Dichloromethane and methanolic extracts of the plant material were tested for activity and found to be effective in inhibiting inflammation. The subfractions obtained by column chromatography were further evaluated for their activities. The active fractions were used to obtain responsible compounds by using semipreparative HPLC. Five coumarin derivatives were isolated and identified as heraclenol (1), byakangelicin (2), heraclenol‐3″‐*O*‐*β*‐glucoside (3), byakangelicin‐3″‐*O*‐*β*‐glucoside (4), and meranzin hydrate III (5). The isolated compounds were investigated for their anti‐inflammatory activities, and heraclenol‐3″‐*O‐β*‐glucoside was found to inhibit the carrageenan and prostaglandin E2‐induced edema significantly compared to the control group and have higher activity than the extracts.

## Graphical abstract

Heraclenol‐3″‐*O*‐*β*‐glucoside, the isolated compound from the roots of *Heracleum sphondylium* subsp. *cyclocarpum* significantly inhibited carrageenan and prostaglandin E2‐induced edema and the activity was identified as being significantly higher than that of the extracts and the control group.

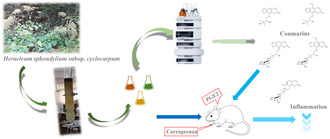

## 1 Introduction

*Heracleum* is one of the largest genera in the Apiaceae family, with 125 species worldwide, especially in Asia. In Turkey, 18 species and 23 taxa are recorded in the flora (Davis 1972; Pimenov and Leonov 2004; Güzel and Kayıkçı 2017; Güneş‐Özkan, Yazlık, and Jaban 2020; TÜBİVES 2023). “Hogweed” and “Tavşancılotu” are the most common names for this genus of plants. These edible plants have been used for many years as a spice and for treating several diseases, including gastrointestinal, cardiovascular, gynecological, cognitive disorders, skin problems, rheumatism, and inflammation (İşcan et al. 2003; Ergene et al. 2006; Dash et al. 2007; Bose et al. 2011; Kousha and Bayat 2012; Bahadori, Dinparast, and Zengin 2016; Park et al. 2017; Zhang et al. 2017). According to folk medicine, *Heracleum* species have been known to alleviate the symptoms of various inflammatory diseases. With their long‐standing history of success, *Heracleum* species offer a natural alternative for treating inflammatory diseases. In Iran Folk Medicine, *H. persicum* Desf. ex Fisch., C.A. Mey. & Avé‐Lall. is used for migraine and sinusitis (Amiri and Joharchi 2013), *H. sphondylium* L. treats rheumatism in South‐Eastern Serbia (Jaric et al. 2015; Vitalini et al. 2015), in India decoction of *H. candolleanum* (Wight et Arn.) Gamble is consumed for nervous and inflammatory diseases, and the essential oil of the roots and fruits of the plant are used as an anti‐inflammatory and aphrodisiac agent respectively (John et al. 2007), *H. moellendorffii* Hance roots treat arthritis, back pain, and fever across Korea, China, and Japan (Bae, Kim, and Lee 2012; Kim et al. 2019), *H. afghanicum* Kitam. leaves are used for fever and pain in Afghanistan (Karimi and Ito 2012) although Traditional Chinese Medicine records the roots of *H. candidans* Wall. ex DC. and *H. yungningense* Hand.‐Mazz. for fever and pain, *H. rapula* Franch. is for pain (Hosseinzadeh, Ramazani, and Razzaghi‐Asl 2019). Lastly, the whole plant of *H. sphondylium* is used against cramps, gastrointestinal disorders, and diarrhea, as reported in Physician's Desk Reference (PDR) monographs (Gruenwald 2000).

Previous studies have evaluated the anti‐inflammatory activities of *Heracleum* species including *H. paphlagonicum* Czeczott, *H. sphondylium* subsp. *ternatum* (Velen.) Brummitt, *H. sphondylium* subsp. *montanum* (Schleich. ex Gaudin) Briq., and *H. sphondylium* subsp. *cyclocarpum* (K. Koch) P.H. Davis using carrageenan, prostaglandin E2 (PGE_2_), and serotonin‐induced hind paw edema test models in vivo. The highest activity was observed with treatment of *H. sphondylium* subsp. *cylocarpum* roots (Kurtul et al. 2025). The current research aims to isolate the active compounds from the roots of *H. sphondylium* subsp. *cyclocarpum*. This will help in obtaining new, anti‐inflammatory structures which could be responsible for the activity of the plant. The new structures may also serve as drug leads for further studies.

## 2 Results

**FIGURE 1:**
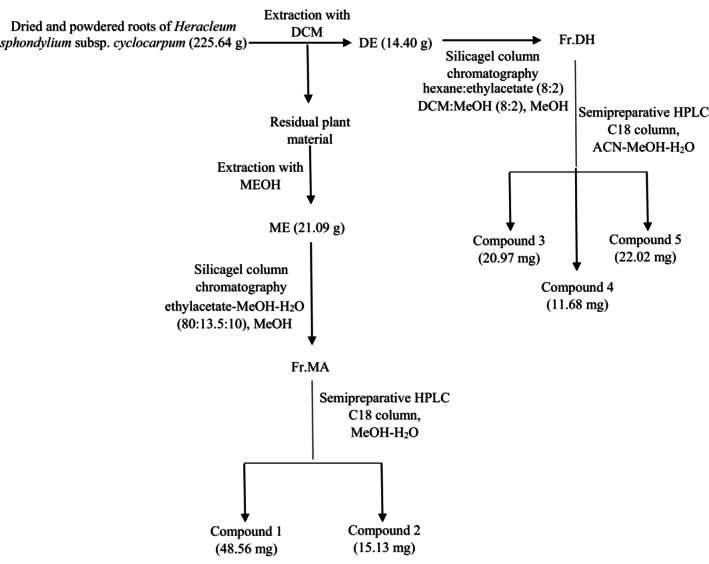
Isolation scheme of the Compound 1–5. ACN, acetonitrile; DCM, dichloromethane; DE, dichloromethane extract; ME, methanolic extract; MeOH, methanol.

**FIGURE 2:**
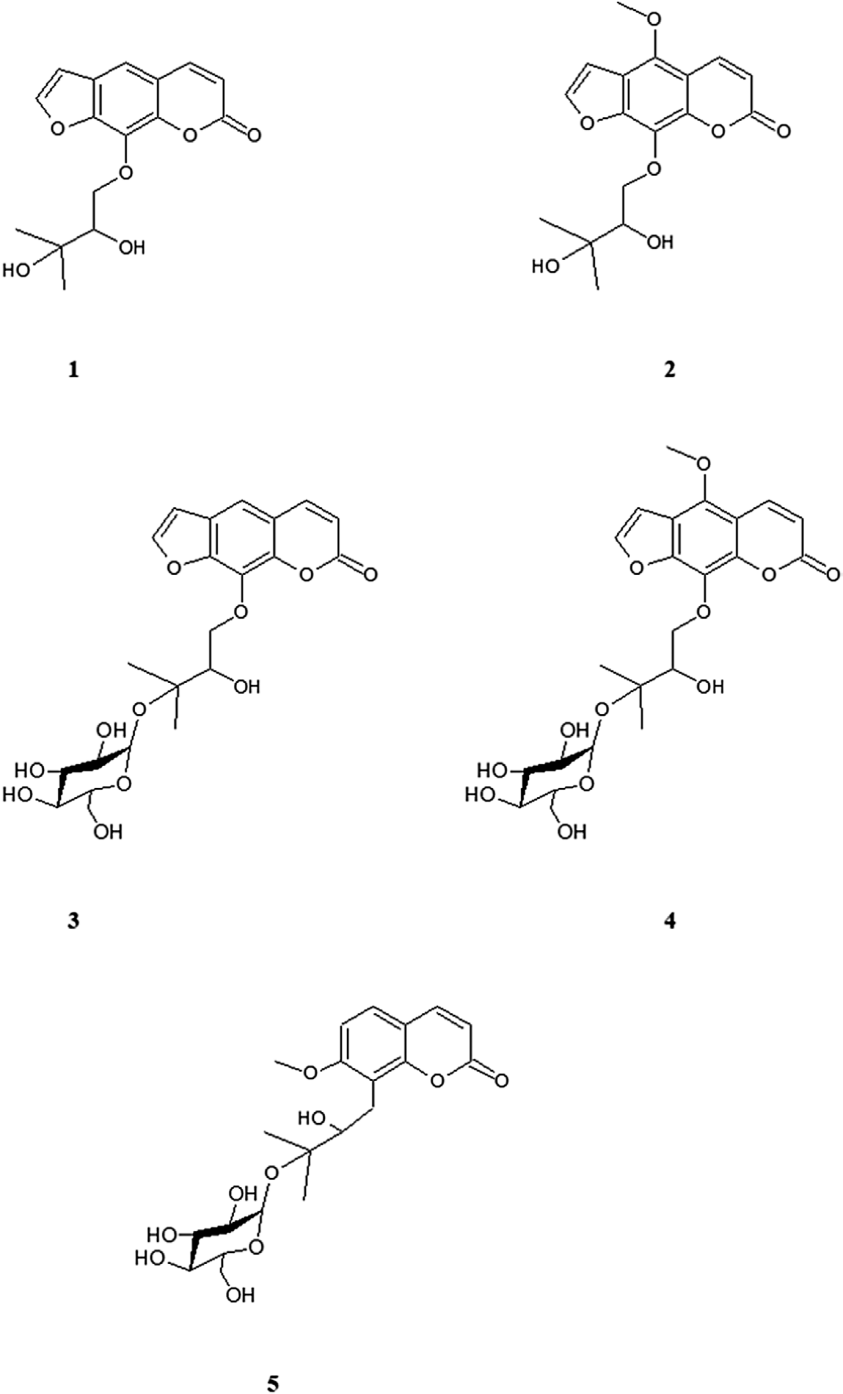
Chemical structures of the isolated compounds (1: heraclenol, 2: byakangelicin, 3: heraclenol‐3″‐*O*‐*β*‐glucoside, 4: byakangelicin‐3″‐*O*‐*β*‐glucoside, 5: meranzin hydrate III).

The present study has revealed that the methanolic and dichloromethane extracts of the roots of *H. spondylium* subsp. *cyclocarpum* has significant anti‐inflammatory properties in various test models. The active fractions of these extracts yielded five different coumarin derivatives (Figure 1), including aglycone and glucoside derivatives (Figure 2). The ^1^H and ^13^C NMR spectra of Compound 1 were revealed the signals observed at δ 6.45 (1H, d, *J* = 9.2), δ 7.75 (1H, d, *J* = 9.2), δ 7.35 (1H, s), 7.68 (1H, d, *J* = 2), 6.80 (1H, d, *J* = 2.4) ppm which are suggested the structure of 8‐substituted furanocoumarin (Table 1). The δ160.62 carbon signal suggested the carbonyl group in the coumarin. By comparison, Compound 1 was determined as heraclenol and literature data confirmed these findings (Niu et al. 2002). On the other hand, Compound 3 (20.97 mg) was obtained as an oily, yellowish substance. The ^1^H and ^13^C NMR spectra of Compound 3 are examined, five proton signals at δ 6.26 (1H, d, *J* = 10), δ 7.90 (1H, d, *J* = 9.6), δ 7.44 (1H, s), δ 7.77 (1H, d, *J* = 2), δ 6.84 (1H, d, *J* = 2) ppm which almost the same as with Compound 1 indicated heraclenol as furanocoumarin structure. Further proton signals were observed at δ 4.37 (1H, dd, *J* = 8; 9, 2) and δ 4.67 (1H, dd, *J* = 2; 8) ppm, and δ 3.93 (1H, dd, *J* = 2, 8) as well as two methyl substituents at δ 1.25 (3H, s) ppm for each, are proved 3‐methyl‐butyl‐1, 2, 3‐ trioxy moiety. Additionally, δ 162.95 ppm carbon signal has confirmed the presence of a carbonyl group in the coumarin ring. The correlation of H‐1″ and C‐8 by HMBC spectrum confirmed heraclenol cycle. The ^13^C NMR spectra displayed at δ 76.56, δ 77.82, δ 80.38, δ 24.93, and δ 24.28 ppm also proved the side chain and based on all obtained data the existence of heraclenol moiety was confirmed. Additionally, a proton at δ 4.45 (1H, d, *J* = 7.6) ppm that belongs to the anomeric carbon placed at δ 98.61 ppm suggests the sugar moiety. All observed proton signals at δ 4.45 (1H, d, *J* = 7.6) as well as δ 3.06–3.71 (6H, m) with carbon signals between δ 98.61 and 62.97 ppm confirmed the glucose moiety. The obtained information was compared with literature data, and the structure of Compound 3 was elucidated as heraclenol‐3″‐*O*‐*β*‐glucoside by comparison of MS measurement, which revealed the compound's molecular weight as m/z 466 (Niu et al. 2002).

**FIGURE 3:**
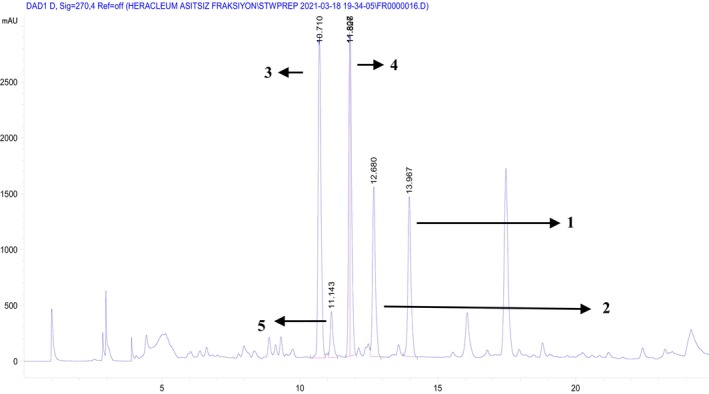
Preparative HPLC chromatogram for the compounds (Respectively, 1: heraclenol, 2: byakangelicin, 3: heraclenol‐3″‐*O*‐*β*‐glucoside, 4: byakangelicin‐3″‐*O*‐*β*‐glucoside, 5: meranzin hydrate III).

**TABLE 1:** ^1^H and ^13^C NMR values of heraclenol and its glucoside.

Chemical shifts (ppm)					
H	Heraclenol	Heraclenol‐3″‐*O*‐*β*‐glucoside	C	Heraclenol	Heraclenol‐3″‐*O*‐*β*‐glucoside
—	—	—	—	—	—
—	—	—	C‐2	160.62	162.95
H‐3	6.43 (1H, d, *J* = 9.2)	6.26 (1H, d, *J* = 10)	C‐3	114.57	115.68
H‐4	7.75 (1H, d, *J* = 9.2)	7.90 (1H, d, *J* = 9,6)	C‐4	144.56	146.93
H‐5	7.35 (1H, s)	7.44 (1H, s)	C‐5	113.59	115.16
—	—	—	C‐6	126.17	128.11
—	—	—	C‐7	147.75	149.48
—	—	—	C‐8	131.59	133.33
—	—	—	C‐9	143.04	144.60
—	—	—	C‐10	116.88	118.11
—	—	—	—	—	—
H‐2′	7.68 (1H, d, *J* = 2)	7.77 (1H, d, *J* =2)	C‐2′	146.85	148.72
H‐3′	6.80 (1H, d, *J* = 2.4)	6.84 (1H, d, *J* =2)	C‐3′	106.85	108.15
H‐1″	4.73 (1H, dd, *J* = 2.8; 10)	4.37 (1H, dd, *J* = 8, 9.2)	C‐1″	75.60	76.56
	4.38 (1H, dd, *J* = 8, 10)	4.67 (1H, dd, *J* = 2.4, 10.4)			
H‐2″	3.87 (1H, dd, *J* = 2.8, 8)	3.93 (1H, dd, *J* = 2, 8)	C‐2″	76.17	77.82
—	—	—	C‐3″	71.59	80.38
H‐4″	1.31 (3H, s)	1.25 (3H, s)	C‐4″	26.45	24.93
H‐5″	1.27 (3H, s)	1.25 (3H, s)	C‐5″	25.03	24.28
H‐1′″	—	4.45 (1H, d, *J* = 7.6)	C‐1′″	—	98.61
H‐2′″	—	3.06 (1H, dd, *J* = 8.0, 9.2)	C‐2′″	—	75.41
H‐3′″	—	3.27 (1H, m)	C‐3′″	—	78.26
H‐4′″	—	3.18 (1H, m)	C‐4′″	—	71.84
H‐5′″	—	3.18 (1H, m)	C‐5′″	—	77.91
H‐6′″	—	3.51 (1H, m)	C‐6′″	—	62.97
		3.71 (1H, dd, *J* = 1.2, 11.2)			

Compound 2 (15.13 mg), obtained from Fr MA by semipreparative HPLC, NMR data revealed that it has almost the same spectral data as the aglycone of Compound 4. The δ 8.20 (1H, d, *J* = 10) and δ 6.25 (1H, d, *J* = 10) proton signals indicate the H‐4 and H‐3 proton of the coumarin structure, and δ 4.19 (3H, s) proton signal represent the 5‐methoxy group. These characteristic signals suggest the structure of 5‐methoxy coumarin. Furan protons of δ 7.81 (1H, d, *J* = 2) and 7.20 (1H, d, *J* = 2.4) considered the linear furanocoumarin structure. δ 4.55 (1H, dd, *J* = 2.8; 10.4); δ 4.26 (1H, dd, *J* = 8, 10.4) ppm proton signals together with two different methyl protons observed at δ 1.26 (3H, s) and δ 1.21 (3H, s) indicate the aliphatic side chain. Further analysis utilizing spectral data suggested that Compound 2, in fact, is the aglycone of Compound 4, leading to its identification as byakangelicin. Structure elucidation of the compound was confirmed through a comparison with the data of byakangelicin, which was previously isolated from *H. platytenium* (Dinçel et al. 2013). Compound 4 (11.68 mg) was isolated from Fr DH; ^1^H and ^13^C NMR findings of Compound 4 are also presented in Table 2. The aglycone part was confirmed as byakangelicin and further signals, δ 4.45 (1H, d, *J* = 7.6) as an anomeric proton and δ 3.05–3.73 (6H, m), suggested that Compound 4 is heteroside of byakangelicin. Observed signals indicated glucose moiety and the linkage of the sugar to the aglycone structure was confirmed by the correlation of anomeric proton signals and the C‐3″. The findings reveal that Compound 4 is byakangelicin‐3″‐*O*‐*β*‐glucoside and is supported by literature data (Dinçel et al. 2013).

**TABLE 2:** ^1^H and ^13^C NMR values of byakangelicin and its glucoside.

^1^H	Chemical shifts δ (ppm)				
	Byakangelicin	Byakangelicin‐3″‐*O*‐*β*‐glucoside	^13^C (ppm)	Byakangelicin	Byakangelicin‐3″‐*O*‐*β*‐glucoside
—	—	—	—	—	—
—	—	—	C‐2	162.71	162.27
H‐3	6.25 (1H, d, *J* = 9.8)	6.18 (1H, d, *J* = 10)	C‐3	113.09	113.30
H‐4	8.20 (1H, d, *J* = 10)	8.13 (1H, d, *J* = 10)	C‐4	141.49	141.66
H‐5	7.35 (1H, s)	—	C‐5	146.08	146.33
—	—	—	C‐6	116.90	116.37
—	—	—	C‐7	151.68	151.94
—	—	—	C‐8	128.33	128.56
—	—	—	C‐9	144.88	145.17
—	—	—	C‐10	108.56	108.75
—	—	—	—	—	—
H‐2′	7.81 (1H, d, *J* = 2.4)	7.72 (1H, d, *J* = 2)	C‐2′	146.99	147.18
H‐3′	7.20 (1H, d, *J* = 2.4)	7.12 (1H, d, *J* = 2.4)	C‐3′	106.47	106.62
H‐1″	4.55 (1H, dd, *J* = 2.4, 10.8)	4.51 (1H, dd, *J* = 2.8, 10.4)	C‐1″	75.60	76.93
H‐2″	4.26 (1H, dd, *J* = 8.0, 10.4)	4.20 (1H, dd, *J* = 8, 10.4)			
—	—	—	C‐2″	76.84	77.81
H‐4″	1.26 (3H, s)	1.24 (3H, s)	C‐3″	72.78	80.39
H‐5″	1.21 (3H, s)	1.24 (3H, s)	C‐4″	26.73	24.22
H‐1‴	—	4.45 (1H, d, *J* = 7.6)	C‐5″	25.09	22.94
H‐2‴	—	3, 05 (1H, dd, *J* = 7.6, 9.2)	C‐1‴	—	98.59
H‐3‴	—	3.17 (1H, m)	C‐2‴	—	75.42
H‐4‴	—	3.17 (1H, m)	C‐3‴	—	78.27
H‐5‴	—	3.17 (1H, m)	C‐4‴	—	71.86
H‐6‴	—	3.73 (1H, dd, *J* = 2.0, 11.6)	C‐5‴	—	77.92
		3.52 (1H, dd, *J* = 5.2, 12.0)	C‐6‴	—	62.99
5‐OCH_3_	4.19 (3H, s)	4.10 (3H, s)	5‐OCH_3_	61.42	79.67

Compound 5 has also been isolated as an oily compound. All ^1^H and ^13^C NMR data are given in Table 3. ^13^C NMR spectra displayed 19 carbon. The presence of a 7‐methoxy 8‐substituted coumarin ring is proved by proton signals at δ 7.76 (1H, d, *J* = 9.2), δ 6.12 (1H, d, *J* = 9.2), δ 7.38 (1H, d, *J* = 8.8), and δ 6.92 (1H, d, *J* = 8.8). A methoxy group is represented by δ 3.83 (3H, s), whereas protons of methyl groups are displayed by δ 1.29 (3H, s) and δ 1.27 (3H, s). Signals at δ 2.83 (1H, dd, *J* = 13.6; 2.4), δ 3.06 (1H, d, *J* = 10.4), and δ 3.68 (1H, dd, *J* = 2.4; 10) suggest the presence of an aliphatic butyl group with two oxygen atoms attached. The anomeric proton and carbon signals at δ 4.45 (1H, d, *J* = 7.6) and δ 98.49 ppm confirmed the presence of glucose as the sugar unit and showed that the anomeric proton of glucose is bound from the *β* position. The compound is identified as meranzin hydrate III, according to literature data (Tian et al. 2019).

**TABLE 3:** ^1^H and ^13^C NMR values of meranzin hydrate III.

Meranzin hydrate III			
H	δ (ppm)	C	δ (ppm)
—	—	—	—
—	—	C‐2	163.84
H‐3	6.12 (1H, d, *J* = 9.2)	C‐3	113.22
H‐4	7.76 (1H, d, *J* = 9.2)	C‐4	146.54
H‐5	7.38 (1H, d, *J* = 8.8)	C‐5	128.65
H‐6	6.92 (1H, d, *J* = 8.8)	C‐6	109.17
—	—	C‐7	162.67
—	—	C‐8	117.29
—	—	C‐9	154.88
—	—	C‐10	114.51
H‐1′	2.83 (1H, dd, *J* = 13.6, 2.4)	C‐1′	26.26
	3.06 (1H, d, *J* = 10.4)		
H‐2′	3.68 (1H, dd, *J* = 2.4, 10)	C‐2′	77.85
—	—	C‐3′	81.60
H‐4′	1.29 (3H, s)	C‐4′	23.54
H‐5′	1.27 (3H, s)	C‐5′	23.40
H‐1″	4.45 (1H, d, *J* = 7.6)	C‐1″	98.49
H‐2″	3.06 (1H, d, *J* = 10)	C‐2″	75.53
H‐3″	3.25 (1H, m)	C‐3″	78.31
H‐4″	3.18 (1H, m)	C‐4″	71.82
H‐5″	3.10 (1H, m)	C‐5″	77.96
H‐6″	3.56 (1H, dd, *J* = 5.2, 12)	C‐6″	62.93
	3.73 (1H, dd, *J* = 1.2, 12)		
7‐OCH_3_	3.83 (3H, s)	7‐OCH_3_	56.91

In the displayed Tables 4, 5, and 6, inhibition levels of extracts, active fractions, and isolated compounds on carrageenan‐induced paw edema are presented. The finding results have revealed that edema was inhibited by DE by 40.5%, 43.4%, and 43.2% after 180, 270, and 360 min, respectively. On the contrary, ME showed inhibition levels of 32.6% and 36.9% at 270 and 360 min, correspondingly. Additionally, it was found that the Fr MA of the ME exhibited inhibition levels of 27.6% and 31.9% after 270 and 360 min, respectively. To determine the specific compounds responsible for their anti‐inflammatory properties, bioactivity‐guided isolation was implemented utilizing the same anti‐inflammatory test models. The two extracts displaying the highest activity were fractionated independently using column chromatography. Finally, the fractions with comparable compositions were combined and assessed utilizing chromatographic techniques. The most active fractions were tested on carrageenan and PGE2‐induced edema, and the most effective ones were determined. Semipreparative HPLC method was used to isolate the effective compounds from these fractions. The following compounds were obtained from fraction Fr DH of the dichloromethane extract: heraclenol‐3″‐*O*‐*β*‐glucoside, byakangelicin 3″‐*O*‐*β*‐glucoside. In addition, methoxy coumarin glucoside meranzin hydrate III was also isolated from the same fraction. Coumarin aglycone compounds, including heraclenol and byakangelicin, furanocoumarin aglycons, were isolated from Fr MA of the methanolic extract. The anti‐inflammatory effects of the isolated compounds were tested using the same anti‐inflammatory activity test models. It was observed that heraclenol‐3″‐*O*‐*β*‐glucoside and byakangelicin 3″‐*O*‐*β*‐glucoside compounds have a significant anti‐inflammatory effect compared to the control group.

**TABLE 4:** Inhibition levels of extracts on carrageenan‐induced paw edema.

Sample	Dose (mg/kg)	Swelling index (x10^−2^mm) ± SEM (inhibition %)			
		90 min	180 min	270 min	360 min
Control		46.21 ± 5.41	53.52 ± 5.92	60.13 ± 5.71	66.81 ± 6.41
DE	100	52.14 ± 3.23	40.51 ± 4.21 (24.3)*	43.41 ± 4.64 (27.8)*	44.22 ± 4.33 (33.8)**
ME	100	51.63 ± 5.13	41.32 ± 4.14 (22.8)	40.51 ± 3.91 (32.6)**	42.13 ± 4.24 (36.9)**
Indomethacin	10	40.31 ± 4.02 (12.8)	39.81 ± 3.70 (25.6)*	38.42 ± 3.52 (36.1)**	37.20 ± 3.41 (44.3)***

Abbreviations: DE, dichloromethane extract; ME, methanolic extract; SEM, standard error meaning.

**TABLE 5:** Inhibition levels of fractions on carrageenan‐induced paw edema.

Sample		Swelling index (×10^−2^mm) ± SEM (inhibition %)				
		Dose (mg/kg)	90 min	180 min	270 min	360 min
Control			41.23 ± 4.70	46.92 ± 4.21	53.62 ± 5.10	59.94 ± 4.83
DE	Fr DA	100	53.42 ± 3.93	59.73 ± 3.84	65.81 ± 4.22	71.42 ± 5.31
	Fr DB	100	44.71 ± 3.82	47.52 ± 3.52	50.42 ± 3.91 (5.9)	52.3 ± 3.42 (12.7)
	Fr DC	100	54.14 ± 5.61	57.64 ± 4.41	59.23 ± 4.61	61.23 ± 4.11
	Fr DD	100	52.34 ± 3.20	53.82 ± 3.70	55.62 ± 4.11	63.42 ± 4.70
	Fr DE	100	53.61 ± 4.14	55.54 ± 4.51	59.11 ± 4.03	62.71 ± 4.31
	Fr DF	100	41.52 ± 3.22	42.40 ± 3.52 (9.6)	44.10 ± 3.90 (17.7)	51.63 ± 3.41 (13.9)
	Fr DG	100	43.54 ± 3.41	46.23 ± 3.82 (1.4)	47.92 ± 3.12 (10.6)	51.51 ± 3.23 (14.0)
	Fr DH	100	42.93 ± 3.53	43.14 ± 3.93 (8.1)	39.60 ± 3.71 (26.1)*	42.42 ± 3.52 (29.2)**
ME	Fr MA	100	42.84 ± 3.81	45.52 ± 3.54 (2.9)	38.82 ± 3.22 (27.6)*	40.82 ± 3.61 (31.9)**
	Fr MB	100	41.72 ± 3.11	46.30 ± 3.72 (1.2)	40.63 ± 4.12 (24.3)	43.11 ± 3.62 (28.0)**
	Fr MC	100	43.54 ± 4.90	45.01 ± 4.51 (4.1)	47.31 ± 4.21 (11.8)	49.62 ± 3.84 (17.2)
	Fr MD	100	42.33 ± 4.14	44.72 ± 4.53 (4.7)	45.22 ± 4.32 (15.7)	50.11 ± 4.43 (16.4)
Indomethacin		10	31.12 ± 3.22 (24.5)*	33.83 ± 3.40 (27.9)**	37.23 ± 3.11 (30.6)**	34.42 ± 3.61 (42.6)***

Abbreviations: DE, dichloromethane extract; ME, methanolic extract; SEM, standard error meaning.

**TABLE 6:** Inhibition levels of isolated compounds on carrageenan‐induced paw edema.

Sample	Dose (mg/kg)	Swelling index (×10^−2^mm) ± SEM (inhibition %)			
		90 min	180 min	270 min	360 min
Control		35.22 ± 4.61	41.01 ± 4.94	48.62 ± 5.10	51.01 ± 4.23
Heraclenol‐3″‐*O*‐*β*‐glucoside	100	31.31 ± 3.21 (11.1)	34.42 ± 2.53 (16.1)	31.61 ± 2.41 (34.9)**	33.80 ± 2.92 (33.7)**
Meranzin hydrate III	100	29.43 ± 3.52 (16.4)	32.71 ± 3.01 (20.2)	36.11 ± 3.72 (25.7)	39.42 ± 3.81 (22.7)
Byakangelicin‐3″‐*O*‐*β*‐glucoside	100	37.10 ± 2.93	40.33 ± 3.12	34.20 ± 3.81 (29.6)**	37.11 ± 3.20 (27.3)*
Heraclenol	100	30.61 ± 5.71 (13.1)	36.32 ± 3.71 (11.5)	38.01 ± 3.22 (21.8)	40.32 ± 3.41 (20.9)
Byakangelicin	100	36.02 ± 4.10	39.61 ± 4.30 (3.4)	40.32 ± 3.90 (17.1)	42.51 ± 3.11 (16.7)
Indometacin	10	24.21 ± 3.31 (31.3)**	30.12 ± 3.12 (26.6)*	35.81 ± 3.21 (26.3)*	31.61 ± 3.02 (38.0)**

Abbreviation: SEM, standard error meaning.

In the study, heraclenol‐3″‐*O*‐*β*‐glucoside was found to be the most effective compound with 34.9% inhibition at minute 270, followed by byakangelicin‐3″‐*O*‐*β*‐glucoside with 29.6% inhibition. Both compounds showed higher activity than indomethacin at minute 270. However, carrageenan‐induced paw edema was not significantly affected by meranzin hydrate III, heraclenol, and byakangelicin.

Regarding the hind paw edema caused by PGE2, it was observed that DE exhibited the strongest activity at minute 45, inhibiting the edema by 33.5%. This inhibition was observed between minutes 30 and 60. Similarly, ME displayed an inhibition range of 26.2% to 35.7% between minutes 30 and 75. Indomethacin activity was determined as significant at minute 30, with the highest inhibition rate of 41.3% being observed at the end of minute 60, as detailed in Table 7.

**TABLE 7:** Inhibition levels of extracts on PGE2‐induced paw edema.

Sample	Dose (mg/kg)	Swelling index (×10^−2^mm) ± SEM (inhibition %)					
		0 min	15 min	30 min	45 min	60 min	75 min
Control		3.74 ± 1.22	16.32 ± 1.53	23.72 ± 1.40	15.83 ± 1.21	12.62 ± 1.32	9.23 ± 1.12
DE	100	3.51 ± 1,03 (5.4)	14.81 ± 1.11 (9.2)	16.41 ± 0.82 (30.8)*	10.51 ± 0.94 (33.5)*	8.73 ± 0.90 (30.9)**	7.14 ± 1.11 (22.8)
ME	100	4.01 ± 0.91	13.63 ± 1.13 (16.6)	17.52 ± 0.81 (26.2)*	11.22 ± 1.03 (29.1)*	8.10 ± 0.81 (35.7)**	6.72 ± 0.73 (27.2)*
Indomethacin	10	36.02 ± 0.52 (2.7)	13.92 ± 1.14 (14.7)	15.21 ± 1.01 (35.9)**	9.91 ± 0.91 (37.3)**	7.42 ± 0.74 (41.3)***	6.11 ± 0.81 (33.7)**

Abbreviations: DE, dichloromethane extract; ME, methanolic extract; SEM, standard error meaning.

The results of the study showed that Fr DH of the DE exhibited 37.3% and 26.9% inhibition in 60 and 75 min, respectively. Fr MA of ME inhibited edema by 31.2%, 39.2%, and 34.3% in 45, 60, and 75 min, respectively. At the end of minute 60, Fr MB of ME exhibited an inhibition rate of only 27.5% for PGE2‐induced paw edema. However, the other fractions of the extracts did not exhibit any significant activity compared to the control group, as indicated in Table 8. Among the isolated compounds, heraclenol‐3″‐*O*‐*β*‐glucoside exhibited the highest activity with a range of 25.3%–38.6% inhibition. Byakangelicin‐3″‐*O*‐*β*‐glucoside exhibited 26.9% inhibition at minute 30 and 27.4% inhibition at minute 45 as a second active compound. The activities of the other compounds were not notable compared to the control group. The inhibition levels of the isolated compounds for PGE2‐induced edema are shown in Table 9.

**TABLE 8:** Inhibition levels of fractions on PGE2‐induced paw edema.

Sample		Swelling index (×10^−2^mm) ± SEM (inhibition %)						
		Dose (mg/kg)	0 min	15 min	30 min	45 min	60 min	75 min
Control			3.14 ± 1.41	12.42 ± 1.61	20.14 ± 1.90	18.93 ± 1.72	15.33 ± 1.50	10.82 ± 1.23
DE	Fr DA	100	3.23 ± 1.01	13.70 ± 1.22	21.32 ± 1.71	19.24 ± 1.91	17.04 ± 1.71	14.41 ± 1.52
	Fr DB	100	3.12 ± 1.22	14.21 ± 1.51	20.72 ± 1.41	21.72 ± 1.10	19.42 ± 1.42	13.24 ± 1.21
	Fr DC	100	3.34 ± 1.54	15.62 ± 1.30	20.93 ± 1.22	22.51 ± 1.61	16.64 ± 1.34	11.74 ± 1.13
	Fr DD	100	3.42 ± 1.71	14.53 ± 1.51	22.64 ± 1.90	20.42 ± 1.52	18.81 ± 1.23	12.43 ± 1.01
	Fr DE	100	3.11 ± 1.13	11.71 ± 1.44 (5.6)	19.42 ± 1.31 (3.5)	17.63 ± 1.71 (6.9)	13.32 ± 1.82 (13.1)	10.91 ± 1.52
	Fr DF	100	3.32 ± 1.42	12.92 ± 1.91	20.81 ± 1.52	16.21 ± 1.42 (14.3)	12.54 ± 1.71 (18.3)	12.34 ± 1.43
	Fr DG	100	3.13 ± 1.21	12.64 ± 1.12	19.61 ± 1.10 (2.5)	16.93 ± 1.22 (10.6)	13.41 ± 1.22 (12.4)	11.23 ± 1.32
	Fr DH	100	3.24 ± 1.62	11.41 ± 1.41 (8.1)	17.11 ± 1.24 (14.9)	14.62 ± 1.41 (22.8)	9.61 ± 1.51 (37.3)*	7.92 ± 1.11 (26.9)*
ME	Fr MA	100	3.44 ± 1.93	10.61 ± 1.80 (14.5)	16.92 ± 1.13 (15.9)	13.01 ± 1.13 (31.2)**	9.32 ± 1.24 (39.2)**	7.11 ± 1.02 (34.3)**
	Fr MB	100	3.23 ± 1.83	10.83 ± 1.73 (12.9)	17.82 ± 1.41 (5.6)	15.32 ± 1.14 (19.0)	11.10 ± 1.13 (27.5)*	11.54 ± 1.41
	Fr MC	100	3.20 ± 1.54	11.91 ± 1.21 (4.0)	18.50 ± 1.62 (7.9)	15.12 ± 1.33 (20.1)	12.21 ± 1.10 (20.3)	8.52 ± 1.53 (21.3)
	Fr MD	100	3.11 ± 1.31	15.72 ± 1.32	18.21 ± 1.21 (9.5)	17.73 ± 1.01 (6.3)	13.22 ± 1.42 (13.7)	11.63 ± 1.64
Indomethacin		10	3.12 ± 1.12	10.12 ± 1.51 (18.5)	15.30 ± 1.70 (23.9)*	12.81 ± 1.21 (32.3)**	8.41 ± 1.10 (45.1)***	6.82 ± 0.92 (37.0)***

Abbreviations: DE, dichloromethane extract; ME, methanolic extract; SEM, standard error meaning.

**TABLE 9:** Inhibition levels of isolated compounds on PGE2‐induced paw edema.

Sample	Dose (mg/kg)	Swelling index (×10^−2^mm) ± SEM (inhibition %)					
		0 min	15 min	30 min	45 min	60 min	75 min
Control		3.53 ± 0.94	11.23 ± 1.82	19.33 ± 1.20	21.51 ± 1.14	14.62 ± 1.01	8.22 ± 0.83
Heraclenol‐3″‐*O*‐*β*‐glucoside	100	4.14 ± 1.42	12.62 ± 1.31	13.91 ± 1.31 (27.9)*	13.21 ± 0.80 (38.6)**	10.91 ± 0.52 (25.3)*	8.51 ± 0.54
Meranzin hydrate III	100	4.01 ± 1.21	15.51 ± 1.44	18.32 ± 1.14 (5.2)	18.12 ± 1.01 (15.8)	16.11 ± 1.31	7.11 ± 0.92 (13.4)
Byakangelicin‐3″‐*O*‐*β*‐glucoside	100	3.52 ± 1.03	10.13 ± 1.03	14.12 ± 1.22 (26.9)*	15.60 ± 1.31 (27.4)*	14.12 ± 1.41 (3.4)	7.42 ± 0.61 (9.8)
Heraclenol	100	3.83 ± 1.14	13.14 ± 1.11	15.02 ± 1.41 (22.3)	17.31 ± 1.12 (19.5)	12.31 ± 1.20 (15.8)	9.01 ± 0.72
Byakangelicin	100	3.92 ± 1.02	11.33 ± 1.12	16.81 ± 1.52 (12.9)	19.40 ± 1.52 (9.8)	15.20 ± 1.72	10.81 ± 0.42
Indomethacin	10	3.54 ± 0.52	9.42 ± 0.71 (16.1)	11.41 ± 1.03 (40.9)***	11.51 ± 1.21 (46.5)***	10.31 ± 1.13 (29.5)*	5.20 ± 0.31 (36.5)**

Abbreviation: SEM, standard error meaning.

Heraclenol‐3″‐*O*‐*β*‐glucoside, byakangelicin‐3″‐*O*‐*β*‐glucoside, meranzin hydrate III, heraclenol, and byakangelicin as isolated compounds in current study from the active fractions of *H. sphondylium* subsp. *cyclocarpum*, have been isolated from various plants belonging to the Apiaceae and Rutaceae families. More particularly *Heracleum* species have been known to contain some of the mentioned structures. For instance, heraclenol is a compound commonly found in the roots of *Heracleum* species such as *H. dissectum* and *H. rapula*. Byakangelicin has also been isolated in the aerial parts of *H. platytaenium* and *H. dissectum*, as well as in the roots of *H. nepalense*. However, previous studies did not detect the presence of byakangelicin 3″‐*O*‐*β* glucoside and meranzin hydrate III in any of the *Heracleum* species. Meranzin hydrate, the aglycone of meranzin hydrate III, was naturally recognized as the synthetic isomer of meranzin until it was first isolated from *Magydaris tomentosa* (Desf.) W.D.J. Koch ex DC flowers (Roselli et al. 2007). Meranzin hydrate has been found in various plants, particularly in the Apiaceae and Rutaceae families. It has been extracted from the roots and rhizomes of *Angelica biserrata* (R.H. Shan and C.Q. Yuan) from the Apiaceae family (Ma et al. 2019), as well as from the leaves of *Phellolophium madagascariense* Baker (Riviere et al. 2006). Other plants that contain meranzin hydrate include *Prangos hulusii* S. G. Şenol, H. YıIdırım and Ö. Seçmen (Şenol, Yıldırım, and Seçmen 2011), *Prangos ferulacea* (L.) Lindl and *Ferulago subvelutina* Rech. F., which have it in their roots (Abshev et al. 1972; Naseri et al. 2013; Tan et al. 2017). Meranzin hydrate has also been isolated from the fruits of *Cnidium monnieri* (L.) Cuss. (Shin et al. 2011), from the aerial parts of *Seseli tortuosum* L.B.S. Eur. (Cecherelli et al. 1990), and from the dried fruit peels of *Citrus aurantium* var. *amara* L. in the Rutaceae family (Sarker et al. 2008). However, none of the *Heracleum* species have been reported to contain meranzin hydrate or its glucosidic forms.

## 3 Discussion

Usage of the *Heracleum* genus plants for their anti‐inflammatory purposes in Chinese Traditional Medicine and in some countries where it is grown is pointed in addition to their other ethnobotanical uses. *Heracleum* genus is one of the widest genera of the Apiaceae family and is distributed in the northern hemisphere (Bahadori, Dinparast, and Zengin 2016). In spite of, there are no bioactivity studies on *H. sphondylium* subsp. *cyclocarpum*, anti‐inflammatory activities of a few *Hearacleum* species have been reported. Hexane extracts of the *H. rapula*, *H. candicans*, *H. moellendorffii*, and *H. stenopterum* roots inhibited the COX‐1 and 5‐LOX in vitro (Liu et al. 1998). Essential oil and hydroalcoholic extract of the *H. persicum* fruits reduced the carrageenan‐induced hind paw edema at 100–200 mg/kg and 400 mg/kg doses, respectively (Hajhashemi, Sajjadi, and Heshmati 2009). No reports have been found about the bioactivities of the isolated compounds of glucosides of byakangelicin and heraclenol. However, previous studies have shown that heraclanol and byakangelicin have some anti‐inflammatory properties. For instance, heraclenol has been found to inhibit the expression of IL‐4, IL‐1β, and TNF‐α at a concentration of 10 μg/mL (Tada et al. 2002). In a study conducted by Garcia‐Argaez et al. (2000), heraclenol was evaluated on mice and observed to reduce TPA‐induced ear edema ranging from 23.1%–75.7% at doses of 0.1–1 mg/ear. On the other hand, byakangelicin has been found to inhibit the expression of various inflammatory markers such as inducible nitric oxide synthase, COX‐2, TNF‐α, and IL‐6. It has also increased collagen and aggrecan levels in mice chondrocytes induced by interleukin (IL)‐1β. These results suggest that byakangelicin could be a potent candidate for treating and preventing osteoarthritis (Zhang et al. 2022). Furthermore meranzin hydrate III is a coumarin glucoside, its aglycone namely meranzin hydrate isolated from *Magydaris tomentosa* (Apiaceae) flowers, has been reported to have antiatherosclerotic activity in mice (Li et al. 2019). Meranzin hydrate was also found to have anti‐inflammatory activity in two different models, xylene‐induced‐ear edema and carrageenan‐induced inflammation, at doses of 1.5 and 3 mg/kg. The study found that meranzin hydrate reduced ear edema by 19.5% and 17.9%, respectively, whereas carrageenan‐induced inflammation was reduced by 37.8% and 47.6%. Furthermore, in vitro tests showed that meranzin hydrate reduced the production of IL‐1β, PGE2, and TNF‐α at a concentration of 5 μg/mL (Zhao et al. 2019).

In this research, the potential of *H. sphondylium* subsp. *cyclocarpum* root extracts were evaluated as an anti‐inflammatory agent. As the *H. sphondylium* subsp. *cyclocarpum* roots showed significant activity on inflammation, bioactivity‐guided isolation procedure was applied to the extracts to determine the active components. The findings revealed that the dichloromethane extract of the roots inhibited carrageenan‐induced hind paw edema and PGE2‐induced inflammation in mice. The significant effect in the carrageenan test after 180 min compared to the control group suggested that the extracts and fractions act on the prostaglandin pathway, not on serotonin. Therefore serotonin was not used to further inflammation assessments. By the bioactivity‐guided fractionation, five coumarin derivatives were isolated, and the isolated compounds were identified as two coumarin aglycones, heraclenol, and byakangelicin, and three furanocoumarin glucosides, heraclenol‐3″‐*O*‐*β*‐glucoside, byakangelicin 3″‐*O*‐*β*‐glucoside, and meranzin hydrate III, a 7‐methoxy coumarin glucoside. Among all the isolated compounds, the glucosides of heraclenol and byakangelicin were found to be active. Specifically, heraclenol‐3″‐*O*‐*β*‐glucoside inhibited the carrageenan and PGE2‐induced edema by 33.7%–34.9% and 25.3%–38.6%, respectively. On the other hand, byakangelicin 3″‐*O*‐*β*‐glucoside showed 27.3%–29.6% and 26.9%–27.4% reduction in carrageenan and PGE2‐induced inflammation, respectively.

## 4 Conclusions

This study is the first to evaluate the anti‐inflammatory properties of the roots of *H. sphondylium* subsp. *cyclocarpum* and isolate its active compounds. This research also discovered meranzin hydrate III in the *Heracleum* genus for the first time. Past studies have mainly focused on the anti‐inflammatory effects of heraclenol and byakangelicin, but no studies have been done on these coumarins' glucoside forms. Furthermore, all studies were performed on these aglycones have been designed in vitro none of the in vivo studies were reported. Through bioactivity‐guided fractionation in this study, it was able to isolate the compound heraclenol‐3″‐*O*‐*β*‐glucoside which exhibited higher activity compared to the extracts and fractions. All of the isolated compounds in the active fractions had a coumarin structure and demonstrated notable anti‐inflammatory activities in test models. This indicates that the coumarin derivatives found in *Heracleum* species could be suggested as responsible for their anti‐inflammatory properties.

Hence, further research is necessary to understand the mechanism of action of heraclenol‐3″‐*O*‐*β*‐glucoside. Additionally, examining standardized extracts based on this compound could provide a potent treatment for inflammation.

## 5 Experimental

### 5.1 General Experimental Procedures

Column chromatography was performed on silica‐gel (Kieselgel 60, 230–400 mesh, 0.040–0.063 mm, Merck). Precoated silica‐gel 60F_254_ plates (Merck 1.05554) were used for thin‐layer chromatography (TLC). High‐Performance Liquid Chromatography (HPLC) analyses and semipreparative HPLC purification were carried out on Agilent 1260 G1315 DAD with C18 column (25 × 4.6 mm; 5 μm).

Waters 2695 Allia Micromass ZQ was used for mass spectrometry. The NMR values were recorded on Varian Mercury 400, 400 MHz High‐Performance Digi NMR spectrometer.

### 5.2 Plant Material

*H. sphondylium* subsp. *cyclocarpum* has been collected from Murgul, Artvin, Turkey (41°14′ 3″ N, 41°33′ 6″ E) in Jul 2019. Plant material was identified by Prof. Dr. Hayri Duman and Prof. Dr. Ahmet Duran. Voucher specimens are kept in the Ankara University Faculty of Pharmacy Herbarium (AEF 28812).

### 5.3 Extraction and Isolation

The plant roots were air‐dried and then ground into a powder weighing 225.64 g. Extraction of the plant material was given in our previous study (Kurtul et al. 2025). Dichloromethane extract (DE) (14.40 g) was subjected to column chromatography. Hexane‐ethylacetate (8:2) and dichloromethane‐methanol (8:2) mixture were used for elution as solvent systems, and methanol (100%) was used for washing column, respectively, and 140 fractions were obtained. The general procedure of the isolation was displayed in Figure 1. Fractions were controlled with TLC developing by hexane‐ethylacetate (8:2; 7:3; 6:4) and ethylacetate‐methanol‐H_2_O (80:13, 5:10) solvent system and by HPLC using acetonitrile‐water (with 0.2% *o*‐phosphoric acid) gradient system. Fractions were combined according to their phytochemical profiles as main eight fractions: Fr DA (1.39 g), Fr DB (1.11 g), Fr DC (1.71 g), Fr DD (0.63 g), Fr DE (0.13 g), Fr DF (0.22 g), Fr DG (7.59 g), Fr DH (1.05 g). Fr DH, which was determined as the most active fraction of DH was separated by semipreparative HPLC with H_2_O (A), acetonitrile (B) and methanol (C) containing gradient solvent system (Figure 3. Exhibits the chromatogram of the isolated compounds). The flow started with 90% A–10% B and changed to 47.5% A–52.5% B up to minutes 20. From the minutes 20.01 to 26, 100% C was passed through the column. The flow rate was 1.5 mL/min, and post time was 4 min. The temperature was used as room temperature, and both UV 210 and 254 nm were used for measurement.

Methanolic extract (ME) of the *H. sphondylium* subsp. *cyclocarpum* roots (21.09 g) was also eluted on silica‐gel column with ethylacetate: methanol‐H_2_O (80:13, 5:10) solvent system to obtain 61 fractions which were investigated by TLC using chloroform‐methanol‐H_2_O (65:25:4) mobile system and by HPLC with acetonitrile‐water (with 0.2% *o*‐phosphoric acid) gradient system. Similar fractions were combined: Fr MA (4.68 g), Fr MB (2.75 g), Fr MC (5.64 g), Fr MD (5.73 g). The active fraction of ME was determined as Fr MA and further separation was performed by semipreparative HPLC, using mobile phase, which made up H_2_O (A), acetonitrile (B) and methanol (C) mixture. The analysis took 21 min, with an additional 4 min of post time. The gradient elution process started with a mixture of A and C solvents in a 70:30 ratio, with a 1.5 mL/min flow rate. At the 5th min, the solvent mixture was changed to A–C (47:53%) and the flow rate was reduced to 1.2 mL/min. At the 11th min, the solvent was changed to A–C (44.3:55.7%). The column was then washed with 100% B for 1 min and 100% C for 6 min, respectively. The isolation was carried out at room temperature.

### 5.4 Structure Elucidation

Mass spectrometry, ^1^H NMR, ^13^C NMR, and 2D NMR (HMBC, COSY, TOCSY, and HSQC) techniques were used to elucidate the structures of the isolated compounds.

### 5.5 Anti‐Inflammatory Activity

#### 5.5.1 Animals

For the tests, it was used male Swiss albino mice weighing between 20 and 25 g purchased from Kobay Animal Breeding Laboratory. They were given pellet food and water ad libitum and were kept in the laboratory for 2 days. Each group consisted of six mice. The study was conducted in accordance with international guidelines for animal experiments and biodiversity rights, as per the ethical standards of the Kobay Animal Breeding Laboratory Ethical Council Project Number: 408.

#### 5.5.2 Preparation of the Test Materials

A solution of 0.5% carboxymethyl cellulose (CMC) was used to suspend the extracts, fractions, and isolated compounds. The mice were administered this solution orally via gastric gavage at a dosage of 100 mg/kg. The control group was given a solution of 0.5% CMC, whereas a reference drug, indomethacin (Nobel), was administered at a dose of 10 mg/kg in 0.5% CMC. After a duration of 60 min, inflammation was induced separately by carrageenan and prostaglandin E2 to observe the effects of the administered materials.

#### 5.5.3 Carrageenan‐Induced Hind Paw Edema

The experiment aimed to induce hind paw edema in mice through an injection of a carrageenan suspension. Each mouse was injected with 25 μL of the suspension, which contained 50 mg of carrageenan from Sigma Co., No: C‐1013, in 2.5 mL saline. For the control group, each mouse received an injection of 25 μL saline solution. The thickness of the right hind paw of each mouse was measured every 90 min for 6 h using a micrometer caliper from Ozaki Co., Tokyo, Japan. This measurement was used to determine the degree of swelling, which is an indicator of inflammation. The difference between the thickness of the right and left hind paws was used as a measure of inflammation. The mean values of each group were compared and analyzed statistically using the methods described previously (Yeşilada and Küpeli 2002; Toker et al. 2004).

#### 5.5.4 PGE2‐Induced Hind Paw Edema

As part of an experiment, a PGE2 solution containing 5 μg of PGE2 (Fluka Chemie AG, Art. 82,475) was injected into the subplantar tissues of the right hind paw of each mouse, whereas the left hind paws were injected with 5 μL of Tyrode's solution. At 15‐min intervals for 75 min, the difference in paw edema between the right and left paws was measured using a micrometer caliper. The mean values of the test and control groups were compared and analyzed statistically, using the methods described by (Yeşilada and Küpeli 2007; Küpeli Akkol and Ercil 2009).

#### 5.5.5 Statistical Analysis of Data

Data from animal experiments were expressed as mean standard error (± SEM). Statistical differences between treatment and control groups were evaluated using ANOVA and Students‐Newman–Keuls post hoc tests. A probability of *p* < 0.05 was considered significant (*p < 0.05; **p < 0.01; ***p < 0.001).

## Conflicts of Interest

The authors declare no conflicts of interest.

## Data Availability

The data that support the findings of this study are available on request from the corresponding author. The data are not publicly available due to privacy or ethical restrictions.
